# Dynamic properties of calcium-activated chloride currents in *Xenopus laevis* oocytes

**DOI:** 10.1038/srep41791

**Published:** 2017-02-13

**Authors:** Ildefonso M. De la Fuente, Iker Malaina, Alberto Pérez-Samartín, María Dolores Boyano, Gorka Pérez-Yarza, Carlos Bringas, Álvaro Villarroel, María Fedetz, Rogelio Arellano, Jesus M. Cortes, Luis Martínez

**Affiliations:** 1 Department of Nutrition, CEBAS-CSIC Institute, Espinardo University Campus, Murcia, Spain; 2 Department of Mathematics, Faculty of Science and Technology, University of the Basque Country, UPV/EHU, Leioa, Spain; 3 Department of Neurosciences, Faculty of Medicine and Dentistry, University of the Basque Country, UPV/EHU, Leioa, Spain; 4 Department of Cell Biology and Histology, Faculty of Medicine and Dentistry, University of the Basque Country, UPV/EHU, Leioa, Spain; 5 Biophysics Unit, CSIC, University of the Basque Country, UPV/EHU, Leioa, Spain; 6 Department of Biochemistry and Pharmacology, Institute of Parasitology and Biomedicine “López-Neyra”, CSIC, Granada, Spain; 7 Laboratory of Cellular Neurophysiology, Neurobiology Institute, UNAM, Querétaro, México; 8 BioCruces Health Research Institute, Cruces University Hospital, Barakaldo, Spain; 9 IKERBASQUE: The Basque Foundation for Science, Bilbao, Spain

## Abstract

Chloride is the most abundant permeable anion in the cell, and numerous studies in the last two decades highlight the great importance and broad physiological role of chloride currents mediated anion transport. They participate in a multiplicity of key processes, as for instance, the regulation of electrical excitability, apoptosis, cell cycle, epithelial secretion and neuronal excitability. In addition, dysfunction of Cl^−^ channels is involved in a variety of human diseases such as epilepsy, osteoporosis and different cancer types. Historically, chloride channels have been of less interest than the cation channels. In fact, there seems to be practically no quantitative studies of the dynamics of chloride currents. Here, for the first time, we have quantitatively studied experimental calcium-activated chloride fluxes belonging to *Xenopus laevis* oocytes, and the main results show that the experimental Cl^−^ currents present an informational structure characterized by highly organized data sequences, long-term memory properties and inherent “crossover” dynamics in which persistent correlations arise at short time intervals, while anti-persistent behaviors become dominant in long time intervals. Our work sheds some light on the understanding of the informational properties of ion currents, a key element to elucidate the physiological functional coupling with the integrative dynamics of metabolic processes.

Chloride (Cl^−^) is thought to be the most abundant free anion in the cell^1^, and its movement through the cellular membranes is mainly mediated by Cl^−^ channels, which seem to be widespread in nearly all cellular organisms, from bacteria to mammals^2^,^3^.

Chloride-conducting anion channels are localized both in the plasma membrane and in intracellular organelles such as the endoplasmic reticulum, the Golgi apparatus, the nucleus, the mitochondria, the lysosomes, the endosomes and the cell vesicles^4^,^5^,^6^,^7^. They participate in a multiplicity of key functions like, for instance, the stabilization of the membrane potential, the regulation of cell volume and electrical excitability, and the acidification of intracellular organelles^4^,^8^. In addition, different studies have recognized the Cl^−^ channels’ contributions to apoptosis^9^, signal transduction^10^, cell cycle^11^, cell adhesion and motility^12^, among other complex cellular processes.

Intracellular chloride currents also play important roles in a variety of physiological processes^13^, including epithelial secretion^14^, neuronal excitability^15^, repolarization of the cardiac action potential^16^, modulation of light responses^17^ and olfactory transduction^18^. It can be noted that, under physiological conditions, certain types of Cl^−^ channels participate in the regulation of the action potentials and synaptic responses, which are important for learning and memory^19^. In fact, dramatic changes in intracellular Cl^−^ currents occur both during development and in response to synaptic activity^20^,^21^.

At a protein metabolism level, there are numerous examples of proteins whose activity is dependent on, or regulated by Cl^− ^22,23,24. For instance, the Na^+^-K^+^-2Cl^−^ cotransporter NKCC1 is activated by low intracellular Cl^−^ via a Cl^−^-sensitive protein kinase^25^.

The importance of chloride channels was also evidenced through studies of human diseases. In fact, the dysfunction of certain types of chloride channels is involved in a variety of diseases such as epilepsy, male infertility, cystic fibrosis, myotonia, lysosomal storage disease, deafness, kidney stones, and osteoporosis^1^,^26^,^27^.

Moreover, different oncogenic processes such as the high rate of proliferation, active migration, and invasiveness of malignant cells into normal tissue have been shown to require the involvement of determined chloride channel activity in a variety of cancer types^22^,^23^.

In general, some chloride channels are activated only by voltage i.e., voltage-gated, while others are activated by various ions e.g., H^+^ (pH), or Ca^2+^, or by the phosphorylation of intracellular residues by several protein kinases^4^,^28^. Based on these and other characteristics, chloride channels have been classified into five main functional groups: (i) extracellular ligand-gated channels, (ii) calcium-activated chloride channels, (iii) volume-regulated anion channels, (iv) cAMP-PKA activated channels, and (v) voltage-gated chloride channels^29^.

Calcium-activated chloride channels (CaCCs) are a key family of chloride channels that regulate the flow of chloride and other monovalent anions across cellular membranes in response to intracellular calcium levels^30^. These channels are ubiquitously expressed, in both excitable and non-excitable cells^31^.

Currents mediated by CaCCs were first observed in 1981 in *Rana pipiens* eggs where the injection of Ca^2+^ initiated a transient shift to positive membrane potentials in a Cl^−^-dependent manner^32^. Later studies in *Xenopus laevis* oocytes and salamander photoreceptors characterized these calcium-activated chloride currents^33^,^34^.

The relationship between chloride currents and intracellular calcium fluctuations gives CaCCs a crucial role in many cellular processes, and numerous studies show the great importance and broad physiological role of these channels^35^.

Historically, chloride channels have been less studied than cation channels. Considerable progress has been made in the knowledge of their molecular structures and functions^30^, but there seems to be practically no quantitative studies of the dynamics of chloride currents. On the contrary, there are a significant number of studies made from the perspective of systems biology on free cations such as calcium. For instance, from the perspective of systems biology, different studies have shown that information might be encoded in the amplitude, the frequency, the duration, the waveform or the timing of the calcium oscillations^36^,^37^. Moreover, the mutual information method was used to calculate the amount of information transferred through a calcium signaling channel^38^ and long-term correlations were also observed in calcium-activated potassium channels^39^.

Here, we present a pioneer quantitative study of the dynamic properties of the chloride currents belonging to calcium-activated chloride channels (CaCCs) of *Xenopus laevis* oocytes, analyzed under different external pH environments (acid, neutral and basic). *Xenopus* oocytes have long been a model system for studying CaCCs because these channels are the predominant channels expressed at extremely high levels (0.5 mA/cm2)^40^.

The calcium-activated chloride currents were measured by the patch-clamp technique and the experimental series were analyzed by means of non-linear approaches. Our main result shows that the currents present a structure characterized by highly organized data sequences, long-term memory and inherent “crossover” dynamics with transitions from persistent to anti-persistent behaviors. In this dynamic structure, short memory time periods with a mean of 7.6 seconds arise from the experimental data, which correspond to non-trivial correlations that encompass around 4,000 experimental chloride values.

In this paper, for the first time, we have addressed essential aspects of calcium-activated chloride channels (CaCCs), and the informational properties herein analyzed seem to be intrinsic characteristics of the dynamics involved in these physiological ion currents.

## Results

**Figure 1:**
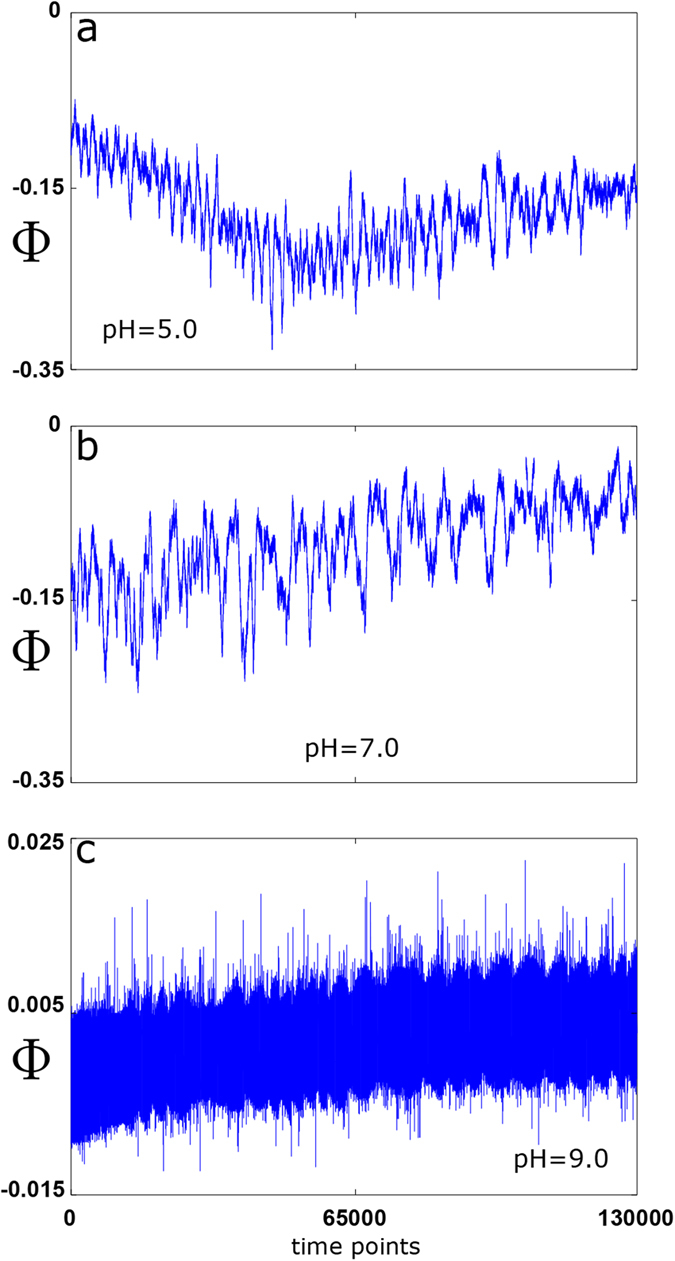
Calcium-activated chloride currents in *Xenopus laevis* oocyte. Three prototype experimental Cl^−^ currents obtained from the same cell at different conditions: (**a**) pH 5.0 (n10), (**b**) pH 7.0 (n11), (**c**) pH 9.0 (n12). Each chloride time series has 130,000 points (sampling interval 2 milliseconds), which correspond to a period of time of 260,000 milliseconds duration. The vertical axis (*Φ*) corresponds to the measures of currents in nanoampers (nA).

In order to study some of the dynamic properties of the chloride channels we have recorded calcium-activated chloride currents in *Xenopus laevis* oocytes, which have been evoked by serum under different external pH stimuli (pH = 0.5, pH = 0.7 and pH = 0.9). Thus, we had 21 time series in total, each one of them formed by 130,000 discrete data points. Figure 1 shows three representative experimental signals obtained by means of the patch-clamp technique, under three different pH conditions, Ringer’s solution at pH 5.0, 7.0 and 9.0 (acid, neutral and basic pH).

**Figure 2:**
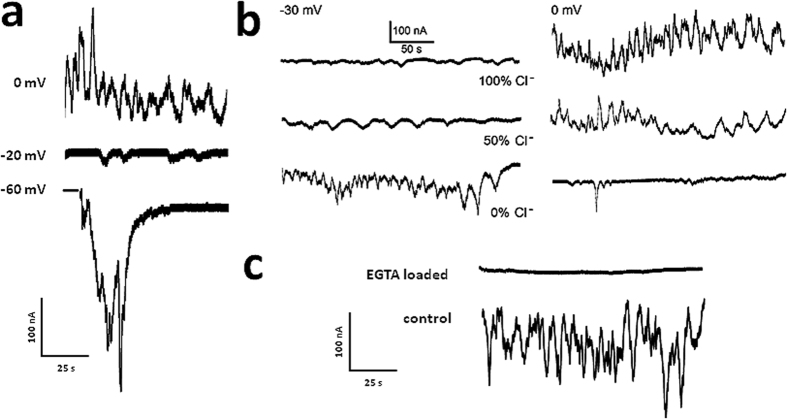
Ca^2+^-dependent Cl^−^ current validation. (**a**) *Xenopus* oocyte held at either −60, −40, −20 or 0 mV. Reversal potential of oscillatory currents corresponded to a value close to −23 mV. (**b**) Oscillatory current reversal potential were dependent on external Cl^−^ concentration, traces show currents in oocytes held at −30 mV or 0 mV in 3 different solutions containing 100%, 50% or 0% Cl^−^, reversal potential shifted toward more positive potentials as external Cl^−^ concentration decreased. (**c**) Cytoplasmic injection of EGTA, a Ca^2+^ chelator, completely eliminated the oscillatory Cl^−^ current.

To confirm that oscillations monitored in *Xenopus* oocytes by application of Fetal Bovine Serum corresponded with Ca^2+^-dependent Cl^−^ currents, three different experiments were performed. First, oocytes generating oscillations were voltage-clamped at 4 different voltages (either −60, −40, −20 or at 0 mV). As it is illustrated in Fig. 2a, currents reversed near to −20 mV, in accordance with the reversal potential of Cl^−^ in oocytes. Second, the reversal potential observed was shifted toward more positive potentials when the external Cl^−^ concentration was reduced, this is shown in Fig. 2b. In this case, oocytes were held to either −30 mV (first column) or 0 mV (second column), while they were superfused with solutions containing 100%, 50% or 0% of Cl^−^ (NaCl was substituted proportionally by Na2SO4 in Ringer solution and, osmolarity compensated adding sucrose). It is clear that reversal potential is close to −30 mV in 100% Cl^−^, while in 0% Cl^−^ oscillations continued being in inward direction at 0 mV, indicating that reversal potential in this condition is more positive. An intermediate case occurs with 50% Cl^−^ solution, where the shift in reversal potential by reducing external Cl^−^ is predicted by the Nernst equation. And finally, it was demonstrated that Cl^−^ currents were Ca^2+^-dependent. Intraoocyte injection of the calcium chelator ethylene glycol-bis(2-aminoethylether)N,N,N’,N’,-tetraacetic acid (EGTA) abolished completely oscillatory currents, according to Ca^2+^-dependent Cl^−^ currents.

First, to test for the presence of long-term correlations in the experimental chloride data we have used the root-mean square (rms) fluctuation *F(l*). For uncorrelated data, the exponent *α* for the relationship *F(l*) ~ *l*^*α*^ is equal to 0.5; in contrast *α* > 0.5 indicates the presence of positive long-range correlations and *α* < 0.5 implies long-term anti-correlations. According to this method, we have divided the 130,000 data points of each time series in 6 non-overlapping windows with *k* = 5, performing the rms fluctuation method on every window for each of the 21 experimental chloride series and fitting *F(l*) within the range *l* = *1*, …, *l*_*max*_ (see Methods for more details). The values of *l*_*max*_ were systematically increased in 100 points, which correspond to 1 second, and the reliability of the rms correlation exponent *α* was calculated by means of the *R*^*2*^ parameter, which measures the *goodness* fit (also called the coefficient of determination).

**Figure 3:**
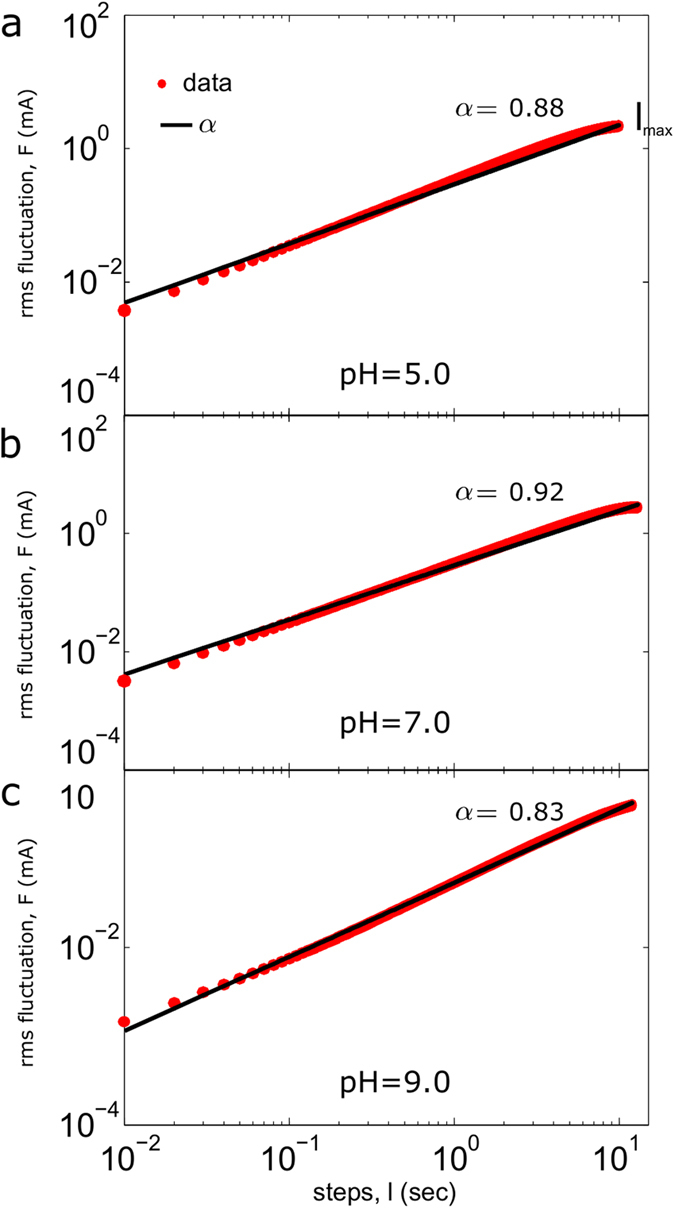
Root mean square fluctuation analysis applied to experiment 1 on a single window. Log-log plot of the rms fluctuation *F* versus *l* step. The red points depict the results of the original data for each value of *l*, while the black lines represent the regression lines. (**a**) *α* = 0.88 (n1), (**b**) *α* = 0.92 (n2) and (**c**) *α* = 0.83 (n3). Corresponding (respectively) *R*^*2*^ adjustment coefficients were 0.9915, 0.9921 and 0.9976. The high values of *α* and *R*^*2*^ indicate non-trivial long-term correlations for each chloride time series during 10, 13 and 12 seconds respectively.

Second, in order to discern whether the experimental Cl^−^ currents exhibit non-trivial correlations, we have fixed a threshold criterion of *R*^*2*^ ≥ 0.99. The obtained *α* values were calculated for every window on each time series, and the results ranged between 0.75 and 1, being 0.927 ± 0.048 (mean ± SD) the global mean 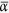 of all the experimental chloride series. These non-trivial correlations encompassed between 1,500 and 6,500 evoked chloride values (mean of 3,809.5 ± 1,298.8), which correspond to periods of time ranging between 3 and 13 seconds (mean of 7.66 ± 2.6). Boundary times where achieved on the series n17 (experiment 6, pH = 7.0) and n2 (experiment 1, pH = 7.0) respectively. The mean rms correlation coefficients (*α*), as well as the number of evoked chloride values under the non-trivial correlation regimen (*N*), with their respective correlation times (*T*_*c*_) for all the experimental series are given in Table 1. Figure 3 shows an example of rms fluctuation analysis applied to three calcium-activated chloride responses of the same oocyte (n1, n2 and n3 time series belonging to the experiment 1) for their *T*_*c*_ times on a single window. In all three cases, the obtained *α* values were significantly different to 0.5, and for at least 10, 13 and 12 seconds respectively, the evoked chloride dynamics presented non-trivial long-term correlations. Alternatively, long term correlations were also observed by calculating the autocorrelation function from the time series (Supplementary Information).

**Figure 4:**
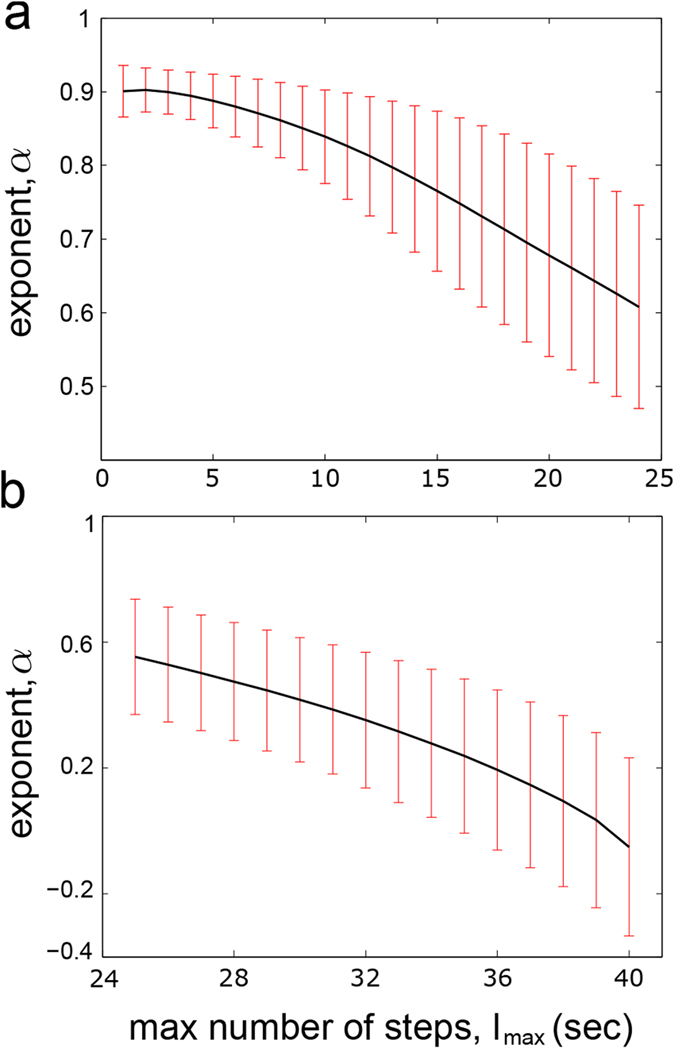
Long-term correlations across different windows lengths. (**a**) Global average 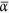 versus different values of *l*_*max*_ (varying from 1 to 24 seconds). (**b**) 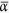 as a function of *l*_*max*_ (varying from 25 to 40 seconds). The error bars represent the standard deviation at each step. It can be observed that all Cl^−^ time series change from positive to negative correlation near *l*_*max*_ = 28 seconds.

Next, we have studied the long-range correlations for *α* ≥ 0.6. The analysis showed exponents ranging between 0.6008 and 0.9718, which respectively correspond to the time series n1 (pH = 5.0, *l*_*max*_ = 2,200) and n17 (pH = 7.0, *l*_*max*_ = 1,200). The global average 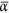 was 0.774 ± 0.108. All the means of *α* values, *R*^*2*^ adjustments, and the *l*_*max*_ are given in Table 2. It can be observed that the values of *α* decrease slowly as *l*_*max*_ increases. This behavior is illustrated in Fig. 4a, where the average 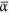 for the 21 time series, as a function of *l*_*max*_, are represented; all the corresponding values of the Fig. 4 are displayed on Table 3.

In addition, we have observed a critical transition around *l*_*max*_ = 28 seconds, where the behavior of the Cl^−^ currents changes from positive to negative correlations (Fig. 4b). It can be observed that as *l*_*max*_ increases, all the *α* exponent values decreased, and for the maximum window length (*l*_*max*_ = 40, corresponding to 20,000 time points), the *α* values were lower than 0.5 (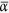 = −0.051 ± 0.283) indicating anti-correlations in all cases; concretely, *α* values ranged between −0.885 and 0.349, which belong to n2 (experiment1, pH = 7.0) and n7 time series (experiment 3, pH = 5.0) respectively.

Finally, we performed a rms fluctuation analysis without the separation of the data in shorter windows, thus considering all the points for each experimental time series, observing anti-correlations for all the cases (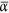 = −0.01 ± 0.1).

Moreover, we have examined whether the chloride currents are described by a fractional Gaussian noise (fGn) or a fractional Brownian motion (fBm) by calculating the slope of the Power Spectral Density plot^41^. The signal exhibits power law scaling if the relationship between its Fourier spectrum and the frequency is approximated asymptotically by *S(f*) ≈ *S(f*_0_)/*f*^*β*^, where *S(f*_*0*_) and *β* are constant values. If −1 < *β* < 1 the signal corresponds to an fGn. In particular, when *β* = *0*, the power spectrum is flat, as is the case for white noise in which the time series is composed of a sequence of independent random values. If 1 < *β* < 3 the signal corresponds to a fBm. The analysis of the Power Spectral Density plot revealed that the experimental series are characterized by a power-law scaling with *β* ranging within 1.507 and 2.991, which suggests that all the series are described by fBm (*β* values are given in Table 4).

Additionally, an analysis of the classical descriptive statistics of the experimental data has been included in the Supplementary Information).

Next, we have checked whether the chloride time series show persistent or anti-persistent long-term memory by calculating the Hurst exponent. Although several tools exist for estimating the long-term memory from fBm time series, one of the most reliable methods is the bridge detrended Scaled Windowed Variance analysis (bdSWV) (see Methods for more details). After bdSWV analysis, the resulting Hurst exponents had a mean value of 0.191 ± 0.101, implying long-range memory and an anti-persistence effect in all the experimental data sets (Table 4). In addition, an ANOVA test revealed that Hurst exponent values were significantly different for time series corresponding to pH = 9.0 in comparison to pH = 7.0 (p-value = 10^−5^) and pH = 5.0 (p-value = 10^−4^), but no significant distinction was found between pH = 7.0 and pH = 5.0 (p-value = 0.42). Notice that the obtained values of H are very low, showing a high degree of anti-persistence (strong trend-reversing), so that an increasing trend in the experimental data values will tend markedly to be followed by a decreasing trend, or a decrease on average will be followed by a robustly increasing trend.

In order to estimate the significance of our results, we have performed a shuffling procedure that defines the null-hypothesis. If the original time series exhibits a memory structure (H ≠ 0.5), after the shuffling such structure will disappear, thus re-applying a new Hurst analysis on the shuffled data should provide values of H close to 0.5. According to this procedure, for each experimental time series (21 in total), we performed a thousand random permutations, which allowed building the null-hypothesis of no correlations. In total, we generated 21,000 random series from the original data belonging to the seven experiments with *Xenopus laevis* oocytes. After shuffling, the results show a mean Hurst exponent of 0.499 ± 0.01, indicating the absence of long-term memory i.e., the informational memory structures in all shuffled series was completely lost. Notice that after shuffling, the series became Gaussian white noise (fGn series with 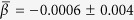, and for this case the use of bdSWV is not justified. Instead, Dispersion Analysis is the most recommendable tool for this kind of series^41^,^42^ (for more details see Methods).

**Figure 5:**
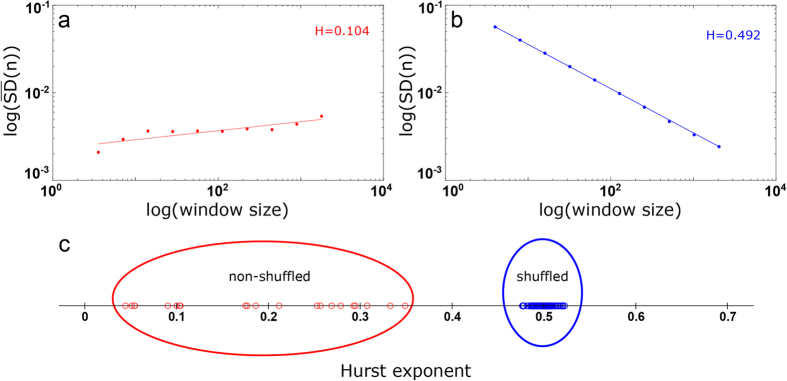
Hurst exponents obtained by the bdSWV analysis. (**a**) The slope of a log-log plot of the 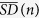 versus the window size for a bdWSV applied to an evoked chloride series (n13, experiment 5, pH = 5.0) gives H = 0.104, indicating the presence of long-term memory. (**b**) The slope of a log-log plot of the *SD(n*) versus the window size for a Dispersion Analysis applied to shuffled time series obtained by randomly permuting all the 130,000 time points for each Cl^−^ time series (n13). After shuffling, H was close to 0.5, indicating the disappearance of the memory structure. (**c**) In red, Hurst exponent values of all the experimental chloride time series; in blue, 100 Hurst exponent values obtained from shuffled series.

Figure 5a illustrates the regression lines of a bdSWV process applied to an example of experimental series giving H = 0.104 (experiment 5, n13, pH = 5.0), which indicates a strong anti-persistent memory. After randomly permuting all the 130,000 points contained in this time series n13, the Dispersion Analysis gave H = 0.492, which indicates a breakdown for the long-term memory (Fig. 5b). In Fig. 5c, we represent 100 Hurst exponent values corresponding to 100 shuffled series, obtained from shuffling the experimental data. It can be observed that, after shuffling, the long-term memory disappears completely in all the time series (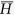 = 0.498 ± 0.01). For illustration purposes, Fig. 5c shows, rather than the 21,000 obtained values of Hurst exponent, only 100 of them. The informational memory structures in all shuffled series were completely broken-down, and therefore, the memory structure that characterizes the experimental data could not be found by chance. Finally, in order to calculate the values of Hurst exponent from short data periods, we used the Detrended Fluctuation Analysis (DFA), because the bdSWV is recommended for data sizes greater than 2^12^, whilst for data sets with less than 2^8^ points bdSWV has been shown to be unreliable^43^. The DFA analysis showed that for time periods ranging between 2 and 5 seconds all the experimental time series exhibit persistent behavior with H > 0.5 being the global mean of 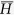 = 0.697 ± 0.11, which indicates that the properties of persistent memory dominate at short time intervals of the calcium-activated chloride currents in *Xenopus laevis* oocytes.

## Discussion

Chloride (Cl^−^) thought to be the most abundant permeable anion in the cell; it participates in a wide variety of important local and systemic physiological processes, while also being involved in a variety of human diseases. Historically, chloride anions have been of less interest than most other free cations. In fact, many molecular aspects of the chloride channels have been well studied, but the characterization of their dynamic properties is still unknown.

Here, we have quantitatively studied experimental Ca^2+^-dependent Cl^−^ currents belonging to *Xenopus laevis* oocytes, which have been evoked by serum under different external pH environments. These Cl^−^ currents were measured by the patch-clamp technique and the data series have been mainly analyzed by means of non-linear dynamic tools.

First, we have applied an analysis based on the root mean square fluctuation and the results revealed non-trivial correlations in all experimental time series. The *α* exponent has a mean of 0.927 (*R*^*2*^ ≥ 0.99) and these strong long-range correlations encompasses concentration values between 1,500 and 6,500, which correspond to time periods ranging between 3 and 13 seconds (with a mean of 7.66 sec). Therefore, the chloride currents present a dynamical structure characterized by long range correlations, and this occurred independently of the experimental condition (here defined by the pH of the cellular external medium).

In addition, transitions from negative to positive correlations were found in the Ca^2+^-dependent Cl^−^ data. Positive long-range correlations arise in short time intervals while negative correlations become dominant over longer ones. This dynamic behavior has been observed in all experimental chloride series.

Moreover, we have calculated the slope of the Power Spectral Density plot concluding that the Cl^−^ data sets can be categorized as fractional Brownian motion i.e., non-stationary series with time-dependent variance.

To test the presence of persistent or anti-persistent memory properties for long time intervals in the experimental data, we have applied the bridge detrended Scaled Windowed Variance analysis, a specific method to obtain Hurst exponent values in fBm signals. We have found that the Hurst exponents satisfy 0.05 < H < 0.35, indicating the existence of anti-persistent long-term memory during long time intervals, in all the series. Values of H < 0.5 have been interpreted as a characteristic for “trend-reversing”, which means that a decreasing trend in the past usually implies an increasing trend (on average) in the future and vice versa, an increase over a set of values in the past is likely to be followed by a decrease in the future.

Our obtained Hurst exponent values are very small (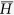 = 0.191 ± 0.101), which shows a high degree of negative dependence between experimental values, indicating strong “trend-reversing”. The strength of this reversion tendency increases as H approaches 0; consequently, when the evoked calcium values spike in one direction, there is a very strong probability that they will subsequently revert back. This important anti-persistent property indicates a self-correcting effect in the experimental data, which describes a situation where tendencies to increase or decrease will tend to reverse themselves.

The high reliability of our Hurst analysis for long time intervals was tested by applying a shuffling procedure (21,000 shuffled time series in total), showing that the Hurst exponent values measured from the original experimental series (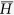 = 0.191 ± 0.101) were significantly different from the ones obtained after shuffling (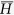 = 0.498 ± 0.01), implying that the correlation structure in all shuffled series was completely broken-down, and therefore, the memory structure that characterizes the original experimental data could not be found by chance.

Finally, in agreement with the observed transitions from negative to positive correlations in the rms fluctuation analysis, we have verified that persistent memory properties arise for short time intervals in all the experimental data sets, while anti-persistent behaviors become dominant in longer intervals. This “crossover phenomenon”, a dynamical property characterized by transitions from persistent to anti-persistent behaviors at a physiological level, seems to show a highly complex regulation of the intracellular chloride currents which exhibit persistence at short time scales (i.e., a trend to increasing in the past will likely be followed by an increasing trend in the future and, vice versa, a trend to decreasing in the past will likely be followed by a decreasing trend in the future), while strong anti-persistence arises in long time scales (when the chloride currents present a determinate trend in the past, there is a high probability to subsequently revert back); this “trend-reversing” behavior suggest that, at long time intervals, the intracellular chloride dynamics are bounded, and reflects the consequences of an inherent self-correcting effect in the system^44^. Similar crossover phenomena have also been observed in some other numerical and experimental physiological processes^44^.

Long-term memory properties found in the calcium-activated chloride behaviors might be related to the dynamic metabolic memory recently proposed to exist in the Cellular Metabolic Structure (CMS in short)^45^,^46^. At a systemic level, cells seem to display a CMS, which behaves as a very complex decentralized information processing system with the capacity to store metabolic memory. According to this framework, the CMS exhibits an essential dynamic informational mechanism by which Hopfield-like attractor dynamics regulate the enzymatic activities. These attractors have the capacity to store functional catalytic patterns that can be correctly recovered by specific input stimuli. The Hopfield-like metabolic dynamics are stable and can be maintained as a long-term functional memory^45^,^46^.

Moreover, since the beginning of the neuronal network modeling of associative memory, the connectivity matrix in the Hopfield network was assumed to result from a long-term memory learning process, occurring over a much slower time scale than neuronal dynamics^47^,^48^,^49^. Therefore, it is well accepted that the attractors emerging in neuronal dynamics described by Hopfield networks are the result of a long-term memory process. Besides, extensive physiological recordings of neuronal processes have revealed the presence of long range correlations in plasticity dynamics for measured synaptic weights. For instance, long tails in the synaptic distribution of weights have been interpreted as short-term memory in neural dynamics^50^.

These studies and others support the thesis that neuronal dynamics exhibit both long-term and short-term memory, and the same may happen with the metabolic processes. In fact, long-term correlations (mimicking short-term memory in neuronal systems) have also been analyzed in different metabolic processes not belonging to the neuronal lineage. One of the most studied is the calcium-activated potassium channels, existing in Leydig cells^51^, kidney Vero cells^52^ and human bronchial epithelial cells^53^. Other biochemical processes also present long-term correlation*s* for example, the intracellular transport pathway of *Chlamydomonas reinhardtii*^54^, the NADPH series of mouse liver cells^55^, and the mitochondrial membrane potential of cardiomyocytes^56^. Similar to what happens in the brain, we believe that the observed long-term memory in the calcium-activated chloride responses might correspond to the short-term memory of the metabolic system involved in these physiological dynamics, and in accordance with our analysis for the non-trivial correlation regimes, this short-term memory could correspond to times around 7 seconds.

In brief, here, we have addressed some essential aspects of calcium- activated chloride currents, in which the concentration dynamics are strongly conditioned by previous concentration measurements over time. Indeed, non-trivial correlations were observed within time-windows of 4,000 experimental concentration values, which correspond approximately to time memory periods with a mean of 7.6 seconds. The analyzed experimental series exhibit fractional Brownian motion, with an informational structure characterized by highly organized data sequences, memory properties and inherent “crossover” dynamics, in which persistent behaviors exist within short time intervals, while anti-persistent dynamics become dominant within long time intervals. In addition, the anti-persistent behavior that encompasses all the points of the time series suggests self-correcting effects in the experimental data. These properties seem to be intrinsic characteristics of the dynamics involved in these physiological processes.

Our work opens up new perspectives for quantitative analysis of the dynamics involved in the dysfunction of calcium-activated chloride channels and sheds some light on the understanding of the informational properties of intracellular signals, a key element to elucidate the physiological functional coupling of the cell with the integrative dynamics of metabolic processes.

## Methods

### Calcium-activated chloride currents in *Xenopus laevis* oocytes

Adult *Xenopus laevis* frogs were obtained from Blades Biological (Cowden, Kent, UK). Oocytes at stage V were plucked from the ovaries and defolliculated by collagenase treatment (type 1, Sigma-Aldrich Quimica, S.A., Madrid, Spain) at 80–630 units/ml in frog Ringer’s solution (115 mM NaCl, 2 mM KCl, 1.8 mM CaCl_2_, 5 mM HEPES at pH 7.0) for 20 min in order to remove the surrounding follicular and epithelial cell layers. Oocytes were maintained at 18 °C in sterile unsupplemented modified Barth’s medium containing (mM): 88 NaCl, 0.2 KCl, 2.4 NaHCO_3_, 0.33 Ca(NO3)_2_, 0.41 CaCl_2_, 0.82 MgSO_4_, 0.88 KH_2_PO_4_, 2.7 Na_2_HPO_4_, with gentamicin 70 μg/ml and adjusted to pH 7.4.

Xenopus oocytes have long been a model system for the study of calcium-activated chloride currents because they express extremely high levels of chloride channels whose activation depends on Ca^2+ ^40.

For this activation we have used Fetal Bovine Serum (FBS). Serum is known to promote oscillations due to alterations of Ca^2+^ concentrations in the cytoplasm, which, as a consequence, evoke Cl^−^ movements across the oocyte membrane^57^ through different calcium-activated chloride channels. According to this procedure, FBS (Sigma-Aldrich) diluted 1:1000 in Ringer’s solution was used for the oocytes’ perfusion to achieve the generation of chloride currents oscillations. The membrane was usually voltage clamped at −60 mV, and in the experiments, three different pH conditions were considered, Ringer’s solution at pH 5.0, 7.0 and 9.0. The sampling interval time scale in the experiments was 2 milliseconds.

All the procedures followed the guidelines of regulation 1201/2005 of Ministerio de Agricultura, Pesca y Alimentacion and the experimental protocols were approved by the University of the Basque Country (UPV/EHU) ethics committee (code: CEBA/8/2009).

### Root mean square fluctuation

An important measure for quantifying long-range correlations in time series is the root mean square (rms) fluctuation^58^, a technique initially developed for random walk studies^59^. Before calculating it, we define the move-step length at time point *i*; here, for the evoked calcium-activated chloride time series, it simply corresponds to electrical current variations, i.e., *u*^*k*^(*i*) ≡ Φ(*i* + *k*) − Φ(*i*) which are given in nanoampers (nA). Without loss of generality, hereon, we denote for a fixed *k, u*^*k*^(*i*) ≡ *u(i*). Next, defining the net displacement after *l* steps as


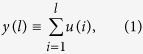


the rms fluctuation of the average displacement is given by:


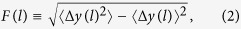


where Δ*y(l*) ≡ *y(l* + *l*_0_) − *y(l*_0_), and brackets denote average over all possible values of *l*_*0*_. Thus, *F(l*) is defined as the square root of the difference between the average of the square of Δ*y(l*) minus the square of its average.

For many processes, *F(l*) scales asymptotically with *l,* i.e., *F(l*) ~ *l*^*α* ^58, and the relationship can be observed by representing *F* as a function of *l* in a log-log plot, fitting *F(l*) in the range *l* = *1*, …, *l*_*max*_. Here, three important regimes can be distinguished, depending on the exponent *α* (rms correlation coefficient)^58^; when *α* = 0.5 the random walk is time uncorrelated and no memory exists. Markov processes initially decay exponentially with *l*, but also give *α* = 0.5 for sufficiently large *l.* If *α* > 0.5, it indicates the presence of positive long-range correlations and *α* < 0.5 implies long-term anti-correlations.

When the method is applied directly to large data sets, there is a risk of concluding that there are no correlations from long-term correlated data. To avoid this issue, data can be subdivided into smaller windows. In our case, the chloride data consisted of 130,000 time points, which we divided into 6 non-overlapping windows of 20,000 points each, leaving the last 10,000 values out of the analysis. The final *α* was calculated averaging over the 6 individual values of *α*, each one calculated within a different window. To estimate the duration of the long-term correlation regime (*T*_*c*_), we increased *l*_*max*_ systematically until the value of *R*^*2*^ (the *goodness* fit in the log-log scale) was smaller than 0.99.

### Hurst exponent Scaled Windowed Variance Analysis

The calculation of the Hurst exponent is a classical method to detect long-term memory in time series introduced by the hydrologist H.E. Hurst in 1951 to study the annual discharges of the Nile River^60^. Afterwards, this method was developed by Mandelbrot in order to apply it to different dynamic processes^61^.

The Hurst exponent, H, is referred to as the index of long-range dependence, which characterizes how the variance depends on a time interval, and also provides information about autocorrelations. The H exponent is also related to the fractal dimension for self-affine series^62^, and for one-dimensional series, H = 2 − D, where D is the fractal dimension and satisfies 1 < D < 2^63^.

The Hurst exponent H satisfies 0 ≤ H ≤ 1. For a random process with independent increments, H is 0.5. When H differs from 0.5, the process is properly fractional and indicates the existence of long-term memory, in which future events have long-term correlations with past events. If H > 0.5, it indicates a biased random process which exhibits persistent behavior. In this case, for several previous transitions, an increment on the average value implies an increasing trend in the future. Conversely, a previously decreasing trend for a sequence of values usually implies a decrease for a similar sequence. Anti-persistent behavior is obtained for 0 ≤ H < 0.5; in this case, a previously decreasing trend implies a probable increasing trend in the future and vice versa, an increase in the past is usually followed by a decrease in the future^41^,^53^. Persistent behavior carries out a superdiffusion, which is faster than in a normal random walk; and, conversely, anti-persistent behavior carries out an abnormal diffusion that is slower than in a normal random walk. In some dynamic processes a transition from persistent to anti-persistent correlation regimes over different time scales, which is known as a “crossover phenomenon”, may emerge^44^.

Two fundamental classes of fractal time series are fractional Brownian motion (fBm) and fractional Gaussian noise (fGn). The fBm is a continuous-time Gaussian process *B*^*H*^(t) with t ≥ 0 such that it satisfies *B*^*H*^(0) = 0 with probability 1, the expectation *E*[*B*^*H*^(t)] is 0 for every t, and the covariance function is given by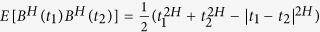 for every t_1_, t_2_ in 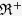, where the parameter H is the Hurst exponent. The fractional Gaussian noise (fGn) is the process *W*^*H*^(*t*), with t ≥ 0, obtained from the fBm increments for discrete time, that is, *W*^*H*^(*t*) = *B*^*H*^(*t* + *1*) − *B*^*H*^(*t*).

The two main, most robust methods to calculate the Hurst exponent are the Dispersion Analysis applied on fractional Gaussian noise (fGn) and Scaled Windowed Variance Analysis for fBm signals^41^.

The Scaled Windowed Variance Analysis (SWVA) is a reliable method for the estimation of the Hurst exponent (H) that has been thoroughly tested on fractional Brownian motion (fBm) signals^43^. In particular, we have used the bridge detrended Scaled Windowed Variance analysis (bdSWV) for the study of calcium-signal time series^41^. To define the SWVA method, let the time series signal be represented by *x*_*t*_, with t = 1, …, *N*, time points. Next, the following steps are carried out for each one of the window sizes *n* = 2, 4, …, *N*/2, *N* (if *N* is not a power of 2, then n takes the values 2, 4, …, 2^*k*^, where *k* is the integer part of log_2_*N*):

1. Partition of the data points in *N/n* adjacent non-overlapping windows {W_1_, …, W_*N/n*_} of size *n*, where W_i_ = {x_(i−1)·*n*+1_, … x_i·*n*_}. If *N* is not a power of 2 and *N* is not divisible by *n*, then the last remaining points are ignored for this value of *n*. For instance, if *N* = 31 and *n* = 4, the first 28 points are partitioned into seven windows.
2. Subtraction of the line between the first and last points in the *n*-th window.
3. For each i = 1, …, *N/n*, calculation of the standard deviation *SD*_*i*_ of the points in each window, by using the formula
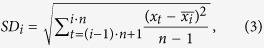
where 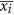 is the average in the window *W*_*i*_.
4. Evaluation of the average 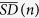 of the *N/n* standard deviations corresponding to equation (3).
5. Observation of the range of the window sizes *n* over which the regression line of log(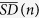) versus log(*n*) gives a good fit (usually some initial and end points are excluded).
6. In this range, the slope of the regression line gives the estimation of the Hurst coefficient H.

Here, to calculate SWVA, we have made use of the program bdSWV, available on the web of the Fractal Analysis Programs of the National Simulation Resource^64^.

### Dispersion Analysis

The Dispersion Analysis (DA) method is applied for the estimation of the Hurst exponent (H) on fractional Gaussian noise (fGn)^42^.

For different bins of length *n*, with *n* varying from 2 to *N*/2, one can define the standard deviation *SD(n*) of the series formed by the mean of the *n* consecutive values of the original series *x*_*i*_. That is, *SD(n*) is the standard deviation of the series *y*_*n,i*_, where


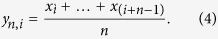


Now, the relation between log(*SD(n*)) and log(*n*) is approximately linear:


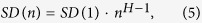


with slope H-1, where H is the Hurst coefficient and *SD*(1) the standard deviation calculated on the first window.

### Detrended Fluctuation Analysis

Detrended Fluctuation Analysis (DFA) is a method that allows for the detection of long-memory processes on non-stationary time series that can be used properly for small data sizes^65^.

The method is summarized as follows: first, given the time series *y*(t) we obtain a signal profile by computing the cumulative sum


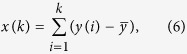


of the time series. Then, the obtained time series is divided into boxes of equal length *n*. Next, the local trend *x*_n_(*k*) in each box is subtracted and the fluctuations of this detrended and integrated signal is calculated by


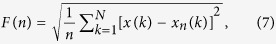


This computation is repeated over all box sizes obtaining a relationship between fluctuations *F(n*) and box sizes *n*. A linear relationship on a log-log graph indicates the presence of scaling, and under such conditions, fluctuations can be characterized by a scaling exponent γ, related to the Hurst exponent^66^. Mainly, if 0 < γ < 0.5, the process is anti-correlated and exhibits anti-persistent behavior, which can be modeled by fGn with H = γ. When 0.5 < γ < 1, the process exhibits positive correlations and persistent behavior which can be modeled by fGn with H = γ, and for a random process with independent increments, γ is 0.5 (H = γ). Other scenarios also can be considered in DFA^66^. Besides, we would like to highlight some of the recent progress in nonlinear time series analysis^67^,^68^,^69^,^70^,^71^.

### Use of experimental cells.

Xenopus laevis frogs (Guy Pluck, Xenopus Express, France) were anaesthetized by hypothermia. Ovary lobules (4–8) were surgically removed under sterile conditions. After surgery, frogs were sutured, and allowed to recover and then returned to housing. No further oocytes were taken for at least 2 months. All the procedures followed the guidelines of regulation 1201/2005 of Ministerio de Agricultura, Pesca y Alimentacion and the experimental protocols were approved by the University of the Basque Country (UPV/ EHU) ethics committee (code: CEBA/8/2009).

## Additional Information

**How to cite this article:** De la Fuente, I. M. *et al*. Dynamic properties of calcium-activated chloride currents in *Xenopus laevis* oocytes. *Sci. Rep.* **7**, 41791; doi: 10.1038/srep41791 (2017).

**Publisher's note:** Springer Nature remains neutral with regard to jurisdictional claims in published maps and institutional affiliations.

## Supplementary Material

Supplementary Information

## Figures and Tables

**Table 1:** The first column shows the number of the experiment, each one corresponding to a single oocyte.

Experiment	Stimulus	Number	*α*	*N*	*T*_*c*_
1	pH5.0	n1	0.9137 ± 0.051	5,000	10
	pH7.0	n2	0.9286 ± 0.009	6,500	13
	pH9.0	n3	0.9118 ± 0.053	6,000	12
2	pH5.0	n4	0.9339 ± 0.035	4,500	9
	pH7.0	n5	0.9177 ± 0.031	5,000	10
	pH9.0	n6	0.9182 ± 0.056	5,000	10
3	pH5.0	n7	0.9226 ± 0.032	3,500	7
	pH7.0	n8	0.9002 ± 0.041	5,500	11
	pH9.0	n9	0.9471 ± 0.078	3,500	7
4	pH5.0	n10	0.9364 ± 0.030	3,500	7
	pH7.0	n11	0.9300 ± 0.037	4,000	8
	pH9.0	n12	0.9295 ± 0.050	2,500	5
5	pH5.0	n13	0.9301 ± 0.036	4,000	8
	pH7.0	n14	0.9199 ± 0.083	4,000	8
	pH9.0	n15	0.9096 ± 0.096	2,000	4
6	pH5.0	n16	0.9420 ± 0.049	3,000	6
	pH7.0	n17	0.9480 ± 0.062	1,500	3
	pH9.0	n18	0.9372 ± 0.049	3,000	6
7	pH5.0	n19	0.9372 ± 0.023	2,500	5
	pH7.0	n20	0.9208 ± 0.021	2,500	5
	pH9.0	n21	0.9433 ± 0.043	3,500	7

The second column contains the pH stimuli applied to each specific experiment. The third one shows the number assigned to each obtained chloride series. The rest of the data corresponds to the values of mean rms correlation coefficient (*α*), number of concentration measurements under the correlation regimen (*N*), and regime correlation time in seconds for non-trivial correlations (*T*_*c*_).

**Table 2:** The first column shows the number of the experiment, each one corresponding to a single oocyte.

Experiment	Stimulus	Number	*α*	*R*^*2*^	max *l*_*max*_
1	pH5.0	n1	0.7094 ± 0.100	0.8896 ± 0.070	2,200
	pH7.0	n2	0.7587 ± 0.092	0.8844 ± 0.120	2,200
	pH9.0	n3	0.7145 ± 0.080	0.8730 ± 0.107	2,300
2	pH5.0	n4	0.7245 ± 0.093	0.8955 ± 0.085	2,200
	pH7.0	n5	0.7638 ± 0.107	0.9243 ± 0.084	2,000
	pH9.0	n6	0.7390 ± 0.103	0.9085 ± 0.059	2,000
3	pH5.0	n7	0.7974 ± 0.089	0.9430 ± 0.084	1,300
	pH7.0	n8	0.7614 ± 0.081	0.9315 ± 0.066	2,000
	pH9.0	n9	0.8474 ± 0.133	0.9721 ± 0.033	1,500
4	pH5.0	n10	0.7918 ± 0.111	0.9288 ± 0.099	1,400
	pH7.0	n11	0.7543 ± 0.128	0.8913 ± 0.113	1,600
	pH9.0	n12	0.7406 ± 0.142	0.9309 ± 0.050	1,500
5	pH5.0	n13	0.7905 ± 0.110	0.9313 ± 0.077	1,500
	pH7.0	n14	0.7792 ± 0.111	0.9551 ± 0.028	1,800
	pH9.0	n15	0.8190 ± 0.136	0.9784 ± 0.034	1,000
6	pH5.0	n16	0.8550 ± 0.119	0.9675 ± 0.061	1,200
	pH7.0	n17	0.8734 ± 0.138	0.9836 ± 0.030	900
	pH9.0	n18	0.7264 ± 0.079	0.9206 ± 0.051	2,000
7	pH5.0	n19	0.7262 ± 0.067	0.9142 ± 0.048	1,300
	pH7.0	n20	0.7837 ± 0.066	0.9435 ± 0.054	900
	pH9.0	n21	0.8137 ± 0.107	0.9549 ± 0.055	1,500

The second column contains the pH stimuli applied to each specific experiment. The third one shows the number assigned to each obtained Cl^−^ series. The rest of the data corresponds to the values of mean rms correlation coefficient (*α*), coefficient of adjustment (*R*^*2*^), and maximum regime correlation points (max *l*_*max*_).

**Table 3:** The first and third columns represent different values of *l*_*max*_, ranging from 1 to 40 seconds.

*l*_*max*_ (sec)	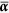	*l*_*max*_ (sec)	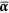
1	0.967 ± 0.04	21	0.647 ± 0.18
2	0.970 ± 0.03	22	0.624 ± 0.18
3	0.966 ± 0.03	23	0.600 ± 0.18
4	0.959 ± 0.04	24	0.577 ± 0.18
5	0.950 ± 0.04	25	0.553 ± 0.18
6	0.939 ± 0.05	26	0.528 ± 0.18
7	0.928 ± 0.06	27	0.502 ± 0.18
8	0.915 ± 0.06	28	0.474 ± 0.18
9	0.901 ± 0.07	29	0.446 ± 0.19
10	0.885 ± 0.08	30	0.416 ± 0.19
11	0.868 ± 0.09	31	0.385 ± 0.20
12	0.849 ± 0.10	32	0.351 ± 0.21
13	0.830 ± 0.11	33	0.316 ± 0.22
14	0.808 ± 0.13	34	0.278 ± 0.23
15	0.786 ± 0.14	35	0.238 ± 0.24
16	0.764 ± 0.15	36	0.193 ± 0.25
17	0.741 ± 0.16	37	0.146 ± 0.26
18	0.717 ± 0.17	38	0.094 ± 0.27
19	0.693 ± 0.17	39	0.034 ± 0.27
20	0.670 ± 0.18	40	−0.051 ± 0.28

The second and forth columns show the values of global mean rms correlation coefficients (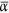) for each *l*_*max*_ values.

**Table 4:** The first column shows the number of the experiment, each one corresponding to a single oocyte.

Experiment	Stimulus	Number	*β*	H
1	pH5.0	n1	2.0248	0.2455 ± 0.0012
	pH7.0	n2	1.9723	0.1744 ± 0.0016
	pH9.0	n3	2.3749	0.0950 ± 0.0019
2	pH5.0	n4	1.8460	0.2501 ± 0.0009
	pH7.0	n5	1.6835	0.3521 ± 0.0007
	pH9.0	n6	1.5669	0.1076 ± 0.0017
3	pH5.0	n7	1.8741	0.1830 ± 0.0012
	pH7.0	n8	1.5075	0.2681 ± 0.0010
	pH9.0	n9	1.7512	0.1071 ± 0.0015
4	pH5.0	n10	1.5834	0.2962 ± 0.0012
	pH7.0	n11	1.6043	0.3174 ± 0.0011
	pH9.0	n12	1.9086	0.0616 ± 0.0023
5	pH5.0	n13	2.0533	0.1040 ± 0.0019
	pH7.0	n14	2.0738	0.1725 ± 0.0016
	pH9.0	n15	1.8572	0.0589 ± 0.0022
6	pH5.0	n16	2.3889	0.3339 ± 0.0009
	pH7.0	n17	2.4520	0.2949 ± 0.0011
	pH9.0	n18	2.1698	0.0526 ± 0.0022
7	pH5.0	n19	2.8441	0.2174 ± 0.0016
	pH7.0	n20	2.5817	0.2718 ± 0.0013
	pH9.0	n21	2.9913	0.0621 ± 0.0026

The second column contains the pH stimuli applied to each experiment. The third one shows the number assigned to each obtained chloride series. The rest of the data corresponds to the values of Power Spectral Density slope (*β*) and Hurst exponent (H) calculated by the bdSWV method.
