# Supplementary material for: Dynamic properties of calcium-activated chloride currents in Xenopus laevis oocytes
Source: Sci Rep. 2017 Feb 13;7:41791. doi: 10.1038/srep41791 (PMC5304176; doi:10.1038/srep41791)
Supplement: Supplementary Information [file srep41791-s1.doc]

**Supplementary Information for: Dynamic properties of calcium-activated chloride currents in *Xenopus laevis* oocytes**

Ildefonso M. De la Fuente^1,2^, Iker Malaina^2^, Alberto Pérez-Samartín^3^, , María Dolores Boyano^4^, Gorka Pérez-Yarza^4^, Carlos Bringas^4^, Álvaro Villarroel^5^, María Fedetz^6^, Rogelio Arellano^7^, Jesus M. Cortes^4,8,9^ and Luis Martínez^2^.

1 Department of Nutrition, CEBAS-CSIC Institute, Espinardo University Campus. Murcia, Spain.

2 Department of Mathematics, Faculty of Science and Technology, University of the Basque Country, UPV/EHU. Leioa, Spain.

3 Department of Neurosciences, Faculty of Medicine and Dentistry, University of the Basque Country, UPV/EHU. Leioa, Spain.

4 Department of Cell Biology and Histology, Faculty of Medicine and Dentistry, University of the Basque Country, UPV/EHU. Leioa, Spain.

5 Biophysics Unit, CSIC, University of the Basque Country, UPV/EHU. Leioa, Spain.

6 Department of Biochemistry and Pharmacology, Institute of Parasitology and Biomedicine “López-Neyra”, CSIC. Granada, Spain.

7 Laboratory of Cellular Neurophysiology, Neurobiology Institute, UNAM. Querétaro, México

8 BioCruces Health Research Institute, Cruces University Hospital. Barakaldo, Spain.

9 IKERBASQUE: The Basque Foundation for Science. Bilbao, Spain.

**Keywords:** chloride currents, long-range correlations, Hurst exponent, calcium-activated chloride channels, time-series analysis.

**Corresponding Author:**

Ildefonso Mtz. de la Fuente Mtz.

CEBAS-CSIC Institute,

Espinardo University Campus,

30100 Murcia, Spain.

E-Mail: mtpmadei@ehu.eus

Tel.: +34-968-396-200; Fax: +34-968-396-213.

**Autocorrelation Analysis**

Calculating the Pearson correlation is a simple way to quantify the statistical dependency between two variables. Values of Pearson correlation lie between -1 and 1. If the value is 1, then the two variables are perfectly correlated, meaning that when one variable increases the other does the same with the same proportion (when one variable is up, the other is up too). The -1 value corresponds to the situation when the two variables are perfectly anti-correlated (when one variable is up, the other is down). If the two variables are statistically independent, the correlation is zero.

Here, we have estimated the Autocorrelation Function (ACF)^S1^, which measures the correlation between *X^p^* and *X^(P+j)^* with *j*=0...*J*. In other words, it measures the cross-correlation of a signal with itself at different time points. The formula for lag k is given by:

$r_{j}=\frac{c_{j}}{c_{0}}$,

where *c_0_* is the sample variance of the time series and

For this analysis, as for the root-mean square fluctuation, the ACF of the step size time series were calculated in 6 non-overlapping windows with *k*=5. Then, the slow decay of the time autocorrelations (normalized between 0 and 1) was quantified by calculating the slope of the ACF function against the lag number in the linear-linear space. The decay duration given in seconds (*T_c_'*, which ranged between 3 and 10 seconds, mean=7.33) was compared with the duration (*T_c_*) of the rms fluctuation analysis (which ranged between 3 and 13 seconds, mean=7.66) by Wilcoxon signed-rank test (a non-parametric test for paired samples). This test indicated that there were no significant differences between the values of these two statistics, with a p-value of 0.4658. All the slopes, the respective *R^2^* adjustments and the *T_c_'* are depicted in the following table:

**Table S1**.

| **Experiment**  **number** | **Stimulus** | **Number** | ***Slope*** | ***R^2^*** | ***T_c_'*** |
| --- | --- | --- | --- | --- | --- |
| 1 | pH5.0 | n1 | -0.0009±0.000 | 0.9559±0.092 | 9 |
|  | pH7.0 | n2 | -0.0010±0.000 | 0.9893±0.005 | 9 |
|  | pH9.0 | n3 | -0.0010±0.001 | 0.9794±0.029 | 10 |
| 2 | pH5.0 | n4 | -0.0009±0.000 | 0.9877±0.014 | 8 |
|  | pH7.0 | n5 | -0.0008±0.000 | 0.9356±0.062 | 8 |
|  | pH9.0 | n6 | -0.0009±0.001 | 0.8327±0.376 | 8 |
| 3 | pH5.0 | n7 | -0.0013±0.001 | 0.9753±0.041 | 7 |
|  | pH7.0 | n8 | -0.0007±0.000 | 0.9037±0.180 | 8 |
|  | pH9.0 | n9 | -0.0010±0.000 | 0.9859±0.019 | 9 |
| 4 | pH5.0 | n10 | -0.0017±0.001 | 0.9954±0.001 | 6 |
|  | pH7.0 | n11 | -0.0018±0.001 | 0.9897±0.004 | 6 |
|  | pH9.0 | n12 | -0.0011±0.001 | 0.9494±0.113 | 9 |
| 5 | pH5.0 | n13 | -0.0017±0.001 | 0.9929±0.005 | 7 |
|  | pH7.0 | n14 | -0.0008±0.000 | 0.8782±0.285 | 9 |
|  | pH9.0 | n15 | -0.0012±0.001 | 0.8746±0.266 | 6 |
| 6 | pH5.0 | n16 | -0.0019±0.001 | 0.9940±0.001 | 6 |
|  | pH7.0 | n17 | -0.0012±0.000 | 0.9855±0.009 | 7 |
|  | pH9.0 | n18 | -0.0014±0.001 | 0.9940±0.002 | 8 |
| 7 | pH5.0 | n19 | -0.0026±0.000 | 0.9927±0.002 | 4 |
|  | pH7.0 | n20 | -0.0031±0.000 | 0.9904±0.002 | 3 |
|  | pH9.0 | n21 | -0.0016±0.001 | 0.9954±0.002 | 7 |

The first column shows the number of the experiment, each one corresponding to a single oocyte. The second column contains the pH stimuli applied to each specific experiment. The third one shows the number assigned to each obtained Cl^-^ series. The rest of the data corresponds to the values of the slope of the ACF against the lag, the respective coefficient of adjustment (*R^2^*) and the duration of the correlation (*T_c_'*) in seconds.

**Descriptive statistics**

The analysis of classical descriptive statistics indicates that the series under pH=9.0 stimulus were found to be significantly different to the ones under pH=5.0 and pH=7.0 in mean, standard deviation and skewness. However, no significant differences were found in these statistics when the experimental series under pH=5.0 and pH=7.0 were compared. Finally, no significant distinction was found in the kurtosis, regardless of the pH stimulus. All the values of mean, standard deviation, skewness and kurtosis are depicted in Table S2:

**Table S2**.

| **Experiment**  **number** | **Stimulus** | **Number** | ***Mean*** | ***SD*** | ***Skewness*** | ***Kurtosis*** |
| --- | --- | --- | --- | --- | --- | --- |
| 1 | pH5.0 | n1 | -0.1562 | 0.0306 | -0.6404 | 3.5040 |
|  | pH7.0 | n2 | -0.0929 | 0.0187 | -0.4287 | 2.7930 |
|  | pH9.0 | n3 | 0.0023 | 0.0042 | 0.0155 | 2.9476 |
| 2 | pH5.0 | n4 | -0.1123 | 0.0275 | -0.8448 | 2.8189 |
|  | pH7.0 | n5 | -0.1808 | 0.0423 | -0.1422 | 2.5152 |
|  | pH9.0 | n6 | -0.0106 | 0.0032 | 0.2777 | 2.8260 |
| 3 | pH5.0 | n7 | -0.0751 | 0.0098 | 0.2337 | 2.9257 |
|  | pH7.0 | n8 | -0.1003 | 0.0194 | -0.4402 | 3.1026 |
|  | pH9.0 | n9 | -0.0085 | 0.0031 | 0.1738 | 2.8967 |
| 4 | pH5.0 | n10 | -0.1973 | 0.0379 | 0.0044 | 2.6504 |
|  | pH7.0 | n11 | -0.1193 | 0.0417 | -0.6329 | 2.9815 |
|  | pH9.0 | n12 | 0.0015 | 0.0037 | 0.3605 | 3.2184 |
| 5 | pH5.0 | n13 | -0.0717 | 0.0099 | -0.0139 | 3.3770 |
|  | pH7.0 | n14 | -0.0819 | 0.0235 | -0.1356 | 2.8191 |
|  | pH9.0 | n15 | -0.0037 | 0.0033 | 0.4442 | 3.1303 |
| 6 | pH5.0 | n16 | -0.2649 | 0.1728 | -0.5498 | 2.4566 |
|  | pH7.0 | n17 | -0.2337 | 0.1128 | -1.1834 | 5.5199 |
|  | pH9.0 | n18 | -0.0309 | 0.0043 | 0.9801 | 4.8203 |
| 7 | pH5.0 | n19 | -0.1521 | 0.0724 | -0.5046 | 2.4556 |
|  | pH7.0 | n20 | -0.2311 | 0.0469 | -0.8855 | 3.8483 |
|  | pH9.0 | n21 | -0.0247 | 0.0061 | -0.3973 | 2.9262 |

The first column shows the number of the experiment, each one corresponding to a single oocyte. The second column contains the pH stimuli applied to each specific experiment. The third one shows the number assigned to each obtained Cl^-^ series. The rest of the data corresponds to the values of the mean, standard deviation (*SD*), skewness and kurtosis of each time series.

**References**

S1. Box, G.E.P., Jenkins, G.M. & Reinsel, G.C. *Time Series Analysis: Forecasting and Control*. (Upper Saddle River, NJ: Prentice-Hall, 1994).
